# 5′-Methyl­sulfanyl-4′-oxo-7′-phenyl-3′,4′-dihydro-1′*H*-spiro­[cyclo­hexane-1,2′-quinazoline]-8′-carbonitrile dimethyl­formamide monosolvate

**DOI:** 10.1107/S1600536811026948

**Published:** 2011-07-13

**Authors:** Xuan Liu, Daxin Shi, Jianhong Tang, Deli Yang, Jiarong Li

**Affiliations:** a School of Chemical Engineering and Environment, Beijing Institute of Technology, Beijing 100081, People’s Republic of China; b College of Chemical Engineering, Huaqiao University, Xiamen Fujian 362021, People’s Republic of China

## Abstract

In the title compound, C_21_H_21_N_3_OS·C_3_H_7_NO, the carbonitrile mol­ecule is built up of two fused six-membered rings and one six-membered ring linked through a spiro C atom. The 1,3-diaza ring adopts an envelope conformation and the cyclo­hexane ring adopts a chair conformation. The dihedral angle between the aromatic rings is 46.7 (3)°. In the crystal, the components are linked by N—H⋯O hydrogen bonds.

## Related literature

For medicinal and biological properties of dihydro­quinazolin-3*H*-4-one derivatives, see: Alagarsamy & Murugesan (2007 ▶); Wang *et al.* (2007 ▶); Jatav *et al.* (2008 ▶); Markosyan *et al.* (2010 ▶). For a related structure, see Zhang *et al.* (2008 ▶).
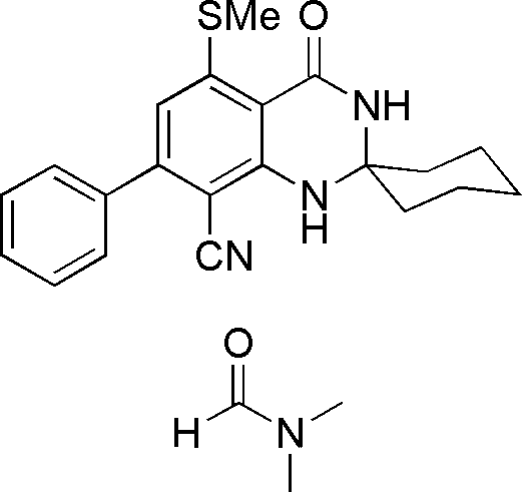


## Experimental

### 

#### Crystal data

C_21_H_21_N_3_OS·C_3_H_7_NO
*M* *_r_* = 436.56
Triclinic, 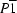

*a* = 9.415 (3) Å
*b* = 10.615 (3) Å
*c* = 12.037 (3) Å
α = 95.092 (3)°
β = 98.156 (4)°
γ = 109.833 (3)°
*V* = 1108.0 (6) Å^3^
*Z* = 2
Mo *K*α radiation
μ = 0.18 mm^−1^
*T* = 153 K
0.31 × 0.31 × 0.12 mm

#### Data collection

Rigaku AFC10/Saturn724+ diffractometer
Absorption correction: ψ scan (North et al., 1968 ▶) *T*_min_ = 0.947, *T*_max_ = 0.979
11758 measured reflections
5754 independent reflections
4274 reflections with *I* > 2σ(*I*)
*R*_int_ = 0.029

#### Refinement

*R*[*F* ^2^ > 2σ(*F* ^2^)] = 0.047
*wR*(*F* ^2^) = 0.117
*S* = 1.00
5754 reflections
291 parameters
H atoms treated by a mixture of independent and constrained refinement
Δρ_max_ = 0.36 e Å^−3^
Δρ_min_ = −0.26 e Å^−3^

### 

Data collection: *CrystalClear* (Rigaku, 2008) ▶; cell refinement: *CrystalClear*; data reduction: *CrystalClear*; program(s) used to solve structure: *SHELXS97* (Sheldrick, 2008 ▶); program(s) used to refine structure: *SHELXL97* (Sheldrick, 2008 ▶); molecular graphics: *SHELXTL* (Sheldrick, 2008 ▶); software used to prepare material for publication: *SHELXL97*.

## Supplementary Material

Crystal structure: contains datablock(s) I, global. DOI: 10.1107/S1600536811026948/aa2012sup1.cif

Structure factors: contains datablock(s) I. DOI: 10.1107/S1600536811026948/aa2012Isup2.hkl

Supplementary material file. DOI: 10.1107/S1600536811026948/aa2012Isup3.cml

Additional supplementary materials:  crystallographic information; 3D view; checkCIF report

## Figures and Tables

**Table 1:** Hydrogen-bond geometry (Å, °)

*D*—H⋯*A*	*D*—H	H⋯*A*	*D*⋯*A*	*D*—H⋯*A*
N1—H11⋯N3^i^	0.838 (10)	2.328 (14)	3.120 (2)	157.96 (3)
N2—H21⋯O1^ii^	0.886 (10)	2.044 (18)	2.927 (3)	174.94 (3)

Symmetry codes: (i) 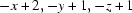; (ii) 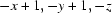.

**Fig. 1.**
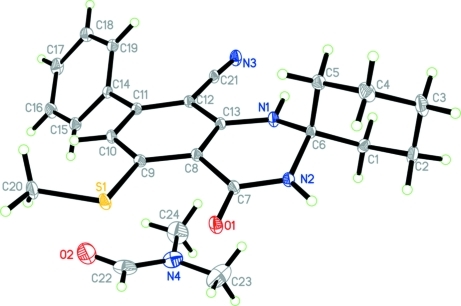
The molecular structure of (I), drawn with 50% probability ellipsoids

**Fig. 2.**
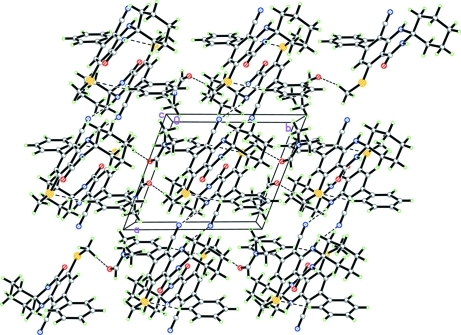
The crystal structure of (I), viewed along a axis

## supplementary crystallographic information

### Comment

The molecular structure of 5'-methylthio-4'-oxo-7'-phenyl-3',4'-dihydro-1'*H*-spiro[cyclohexane-1, 2'-quinazoline]-8'-carbonitrile(I) is built up with one six membered ring and two fused six membered rings and a six membered ring linked through a spiro C atom (Fig. 1). The ring of C1, C2, C3, C4, C5, C6 exists in an chair conformation, and the C6 atom displaced by 0.647 (6)Å from the ring of C1, C2, C4, C5 (planar within 0.019 (5) Å), while C3 atom displaced by 0.669 (8) Å. The 1,3-diaza ring exists in an envelope conformation, and the C6 atom displaced by 0.476 (5)Å from the rest of the atoms of the 1,3-diaza ring (planar within 0.013 (5) Å). The dihedral angle between the plane (C14, C15, C16, C17, C18, C19) and the plane (C8, C9, C10, C11, C12, C13) is 46.7 (3)°. The angle between C9, S1, C20 is 102.83 (8) Å. The geometry of the fused rings compares well with the related spiro[cyclopentane-1,2'(1'*H*)-quinazolin]-4'(3'*H*)-one] (Zhang *et al.*, 2008). The crystal packing is stabilized by intermolecular N—H···O and N—H···N bonds.

### Experimental

To a solution of cyclohexanone (3 ml) and sodium hydroxide (0.3 mmol) were added 3-amino-5-(methylthio)biphenyl-2,4-dicarbonitrile (0.1 mmol). The mixture was heated at 373 K for 2.0 h. The reaction mixture was cooled with ice water (20 ml) and then filtered to give the title compound. The product was recrystallizated from a mixed solvent (ethanol:*N*,*N*-dimethylformamide 1:3) to obtain colorless single crystals. M.p. 572–573 K. Spectra data: IR (KBr): 3317, 3219, 2933, 2216, 1650, 1585, 1550, 1413, 771, 709 cm^-1^. ^1^H-NMR (DMSO-d_6_, p.p.m.): 0.92–1.84 (10*H*, m, C_5_H_10_), 2.38 (3*H*, s, SCH_3_), 6.50(1*H*, s, NH), 6.61 (1*H*, s, ArH), 7.50–7.61 (5*H*, m, ArH), 8.29 (1*H*, s, NH). ESI-MS m/*z*: [*M*+H]^+^ 364.1. C_21_H_21_N_3_OS: calcd. % C 69.39, H 5.82, N 11.56; found % C 69.28, H 5.68, N 11.31.

### Refinement

H atoms bonded to C atoms were included in the calculated positions and refined as riding with *U*_iso_(H)=1.2*U*_eq_(C) or 1.5*U*_eq_(*C*)(methyl). H atoms of NH group were located in difference Fourrier maps and refined inependently with *U*_iso_(H)=1.5*U*_eq_(N).

### Crystal data

**Table:**

C_21_H_21_N_3_OS·C_3_H_7_NO	*Z* = 2
*M**_r_* = 436.56	*F*(000) = 464
Triclinic, *P*1	*D*_x_ = 1.309 Mg m^−^^3^
Hall symbol: -P 1	Mo *K*α radiation, λ = 0.71073 Å
*a* = 9.415 (3) Å	Cell parameters from 3507 reflections
*b* = 10.615 (3) Å	θ = 2.5–29.1°
*c* = 12.037 (3) Å	µ = 0.18 mm^−^^1^
α = 95.092 (3)°	*T* = 153 K
β = 98.156 (4)°	Chunk, colorless
γ = 109.833 (3)°	0.31 × 0.31 × 0.12 mm
*V* = 1108.0 (6) Å^3^	

### Data collection

**Table:**

Rigaku AFC10/Saturn724+ diffractometer	5754 independent reflections
Radiation source: Rotating Anode	4274 reflections with *I* > 2σ(*I*)
graphite	*R*_int_ = 0.029
Detector resolution: 28.5714 pixels mm^-1^	θ_max_ = 29.1°, θ_min_ = 2.5°
phi and ω scans	*h* = −12→12
Absorption correction: ψ scan (North et al., 1968)	*k* = −12→14
*T*_min_ = 0.947, *T*_max_ = 0.979	*l* = −16→15
11758 measured reflections	

### Refinement

**Table:**

Refinement on *F*^2^	Primary atom site location: structure-invariant direct methods
Least-squares matrix: full	Secondary atom site location: difference Fourier map
*R*[*F*^2^ > 2σ(*F*^2^)] = 0.047	Hydrogen site location: inferred from neighbouring sites
*wR*(*F*^2^) = 0.117	H atoms treated by a mixture of independent and constrained refinement
*S* = 1.00	*w* = 1/[σ^2^(*F*_o_^2^) + (0.0568*P*)^2^ + 0.160*P*] where *P* = (*F*_o_^2^ + 2*F*_c_^2^)/3
5754 reflections	(Δ/σ)_max_ = 0.001
291 parameters	Δρ_max_ = 0.36 e Å^−^^3^
0 restraints	Δρ_min_ = −0.26 e Å^−^^3^

### Special details

**Table:**

Geometry. All s.u.'s (except the s.u. in the dihedral angle between two l.s. planes) are estimated using the full covariance matrix. The cell s.u.'s are taken into account individually in the estimation of s.u.'s in distances, angles and torsion angles; correlations between s.u.'s in cell parameters are only used when they are defined by crystal symmetry. An approximate (isotropic) treatment of cell s.u.'s is used for estimating s.u.'s involving l.s. planes.
Refinement. Refinement of *F*^2^ against ALL reflections. The weighted *R*-factor *wR* and goodness of fit *S* are based on *F*^2^, conventional *R*-factors *R* are based on *F*, with *F* set to zero for negative *F*^2^. The threshold expression of *F*^2^ > σ(*F*^2^) is used only for calculating *R*-factors(gt) *etc*. and is not relevant to the choice of reflections for refinement. *R*-factors based on *F*^2^ are statistically about twice as large as those based on *F*, and *R*- factors based on ALL data will be even larger.

### Fractional atomic coordinates and isotropic or equivalent isotropic displacement parameters (Å^2^)

**Table:**

	*x*	*y*	*z*	*U*_iso_*/*U*_eq_	
S1	0.30973 (5)	0.65929 (4)	0.27438 (3)	0.02075 (11)	
O1	0.43889 (14)	0.57074 (13)	0.11197 (9)	0.0275 (3)	
N4	0.75021 (19)	0.94951 (17)	0.28720 (14)	0.0374 (4)	
O2	0.6020 (2)	1.02046 (18)	0.39412 (16)	0.0580 (5)	
N1	0.79059 (16)	0.52496 (15)	0.31427 (11)	0.0197 (3)	
N2	0.63848 (16)	0.49735 (14)	0.13547 (11)	0.0200 (3)	
N3	0.99764 (17)	0.62325 (16)	0.58365 (11)	0.0259 (3)	
C1	0.87655 (18)	0.44768 (18)	0.15077 (13)	0.0221 (3)	
H1A	0.9264	0.5415	0.1362	0.026*	
H1B	0.9511	0.4266	0.2057	0.026*	
C2	0.8378 (2)	0.3509 (2)	0.03980 (14)	0.0288 (4)	
H2A	0.7780	0.3812	−0.0195	0.035*	
H2B	0.9341	0.3531	0.0145	0.035*	
C3	0.7457 (2)	0.2069 (2)	0.05401 (16)	0.0362 (5)	
H3A	0.8102	0.1724	0.1062	0.043*	
H3B	0.7158	0.1481	−0.0204	0.043*	
C4	0.6018 (2)	0.20061 (19)	0.10126 (16)	0.0316 (4)	
H4A	0.5451	0.1063	0.1114	0.038*	
H4B	0.5338	0.2292	0.0468	0.038*	
C5	0.64463 (19)	0.29280 (17)	0.21494 (14)	0.0226 (3)	
H5A	0.7069	0.2603	0.2707	0.027*	
H5B	0.5499	0.2887	0.2436	0.027*	
C6	0.73564 (17)	0.43950 (16)	0.20362 (12)	0.0166 (3)	
C7	0.54013 (18)	0.54868 (16)	0.17623 (13)	0.0187 (3)	
C8	0.56754 (17)	0.58920 (16)	0.30038 (12)	0.0163 (3)	
C9	0.47755 (17)	0.65007 (16)	0.35316 (13)	0.0166 (3)	
C10	0.52394 (17)	0.70489 (16)	0.46842 (13)	0.0178 (3)	
H10	0.4628	0.7455	0.5037	0.021*	
C11	0.65724 (17)	0.70114 (15)	0.53231 (13)	0.0165 (3)	
C12	0.74383 (17)	0.63548 (16)	0.48131 (12)	0.0160 (3)	
C13	0.69977 (17)	0.57954 (15)	0.36522 (13)	0.0160 (3)	
C14	0.71051 (17)	0.77401 (16)	0.65038 (13)	0.0168 (3)	
C15	0.70835 (19)	0.90471 (17)	0.67204 (14)	0.0222 (3)	
H15	0.6717	0.9444	0.6119	0.027*	
C16	0.7592 (2)	0.97693 (18)	0.78055 (14)	0.0276 (4)	
H16	0.7583	1.0662	0.7942	0.033*	
C17	0.8112 (2)	0.9198 (2)	0.86898 (14)	0.0303 (4)	
H17	0.8468	0.9700	0.9431	0.036*	
C18	0.8114 (2)	0.7890 (2)	0.84928 (14)	0.0282 (4)	
H18	0.8453	0.7491	0.9103	0.034*	
C19	0.76196 (19)	0.71616 (18)	0.74030 (13)	0.0218 (3)	
H19	0.7632	0.6270	0.7270	0.026*	
C20	0.2291 (2)	0.72814 (19)	0.38122 (15)	0.0271 (4)	
H20A	0.2100	0.6684	0.4393	0.033*	
H20B	0.1321	0.7352	0.3461	0.033*	
H20C	0.3013	0.8182	0.4163	0.033*	
C21	0.88385 (18)	0.63108 (16)	0.54123 (13)	0.0183 (3)	
C22	0.6208 (3)	0.9699 (2)	0.3065 (2)	0.0446 (6)	
H22	0.5366	0.9415	0.2448	0.053*	
C24	0.8796 (3)	0.9892 (3)	0.3788 (2)	0.0567 (7)	
H24A	0.8536	1.0275	0.4468	0.068*	
H24B	0.9676	1.0573	0.3576	0.068*	
H24C	0.9058	0.9099	0.3944	0.068*	
C23	0.7636 (3)	0.8852 (3)	0.1816 (2)	0.0654 (8)	
H23A	0.6646	0.8547	0.1295	0.078*	
H23B	0.7935	0.8070	0.1947	0.078*	
H23C	0.8418	0.9496	0.1485	0.078*	
H11	0.855 (2)	0.504 (2)	0.3564 (16)	0.029 (5)*	
H21	0.621 (2)	0.4760 (19)	0.0607 (17)	0.030 (5)*	

### Atomic displacement parameters (Å^2^)

**Table:**

	*U*^11^	*U*^22^	*U*^33^	*U*^12^	*U*^13^	*U*^23^
S1	0.01842 (19)	0.0274 (2)	0.0198 (2)	0.01458 (16)	0.00019 (15)	0.00021 (16)
O1	0.0315 (7)	0.0441 (8)	0.0155 (6)	0.0282 (6)	−0.0021 (5)	−0.0003 (5)
N4	0.0314 (9)	0.0385 (10)	0.0354 (9)	0.0042 (7)	0.0027 (7)	0.0091 (8)
O2	0.0584 (11)	0.0559 (11)	0.0747 (12)	0.0312 (9)	0.0266 (10)	0.0201 (10)
N1	0.0200 (7)	0.0298 (8)	0.0127 (6)	0.0164 (6)	−0.0019 (5)	−0.0024 (6)
N2	0.0241 (7)	0.0299 (8)	0.0117 (7)	0.0179 (6)	0.0011 (5)	0.0016 (6)
N3	0.0253 (7)	0.0387 (9)	0.0182 (7)	0.0192 (7)	0.0016 (6)	−0.0002 (6)
C1	0.0196 (7)	0.0350 (10)	0.0168 (8)	0.0153 (7)	0.0048 (6)	0.0058 (7)
C2	0.0305 (9)	0.0491 (12)	0.0175 (8)	0.0277 (9)	0.0062 (7)	0.0020 (8)
C3	0.0482 (12)	0.0418 (12)	0.0254 (10)	0.0303 (10)	0.0008 (8)	−0.0086 (8)
C4	0.0362 (10)	0.0254 (10)	0.0309 (10)	0.0125 (8)	0.0003 (8)	−0.0041 (8)
C5	0.0231 (8)	0.0256 (9)	0.0215 (8)	0.0118 (7)	0.0042 (6)	0.0029 (7)
C6	0.0189 (7)	0.0239 (8)	0.0104 (7)	0.0130 (6)	0.0013 (6)	0.0006 (6)
C7	0.0210 (7)	0.0224 (8)	0.0156 (8)	0.0126 (6)	0.0016 (6)	0.0009 (6)
C8	0.0169 (7)	0.0198 (8)	0.0141 (7)	0.0095 (6)	0.0014 (6)	0.0013 (6)
C9	0.0155 (7)	0.0187 (8)	0.0163 (7)	0.0076 (6)	0.0014 (6)	0.0031 (6)
C10	0.0182 (7)	0.0215 (8)	0.0170 (8)	0.0112 (6)	0.0044 (6)	0.0015 (6)
C11	0.0172 (7)	0.0178 (8)	0.0154 (7)	0.0072 (6)	0.0036 (6)	0.0023 (6)
C12	0.0166 (7)	0.0185 (8)	0.0142 (7)	0.0087 (6)	0.0010 (6)	0.0015 (6)
C13	0.0167 (7)	0.0176 (8)	0.0158 (7)	0.0087 (6)	0.0032 (6)	0.0022 (6)
C14	0.0162 (7)	0.0209 (8)	0.0145 (7)	0.0083 (6)	0.0039 (6)	0.0006 (6)
C15	0.0251 (8)	0.0260 (9)	0.0182 (8)	0.0131 (7)	0.0042 (6)	0.0014 (7)
C16	0.0341 (10)	0.0251 (9)	0.0253 (9)	0.0147 (8)	0.0059 (7)	−0.0048 (7)
C17	0.0343 (10)	0.0404 (11)	0.0159 (8)	0.0176 (8)	0.0002 (7)	−0.0078 (7)
C18	0.0320 (9)	0.0418 (11)	0.0165 (8)	0.0213 (8)	0.0022 (7)	0.0046 (8)
C19	0.0248 (8)	0.0260 (9)	0.0189 (8)	0.0136 (7)	0.0063 (6)	0.0043 (7)
C20	0.0240 (8)	0.0369 (11)	0.0274 (9)	0.0203 (8)	0.0055 (7)	0.0013 (8)
C21	0.0229 (8)	0.0220 (8)	0.0121 (7)	0.0117 (6)	0.0032 (6)	−0.0017 (6)
C22	0.0348 (11)	0.0402 (13)	0.0534 (14)	0.0054 (9)	0.0004 (10)	0.0248 (11)
C24	0.0370 (12)	0.0674 (18)	0.0595 (16)	0.0165 (12)	−0.0021 (11)	0.0040 (13)
C23	0.079 (2)	0.0561 (17)	0.0461 (15)	0.0015 (14)	0.0222 (14)	0.0024 (12)

### Geometric parameters (Å, °)

**Table:**

S1—C9	1.7611 (16)	C8—C9	1.411 (2)
S1—C20	1.8033 (17)	C8—C13	1.412 (2)
O1—C7	1.2394 (18)	C9—C10	1.402 (2)
N4—C22	1.355 (3)	C10—C11	1.391 (2)
N4—C23	1.431 (3)	C10—H10	0.9500
N4—C24	1.437 (3)	C11—C12	1.407 (2)
O2—C22	1.204 (3)	C11—C14	1.486 (2)
N1—C13	1.3655 (19)	C12—C13	1.414 (2)
N1—C6	1.4616 (19)	C12—C21	1.428 (2)
N1—H11	0.84 (2)	C14—C15	1.396 (2)
N2—C7	1.349 (2)	C14—C19	1.398 (2)
N2—C6	1.4644 (19)	C15—C16	1.385 (2)
N2—H21	0.89 (2)	C15—H15	0.9500
N3—C21	1.152 (2)	C16—C17	1.381 (3)
C1—C2	1.529 (2)	C16—H16	0.9500
C1—C6	1.531 (2)	C17—C18	1.389 (3)
C1—H1A	0.9900	C17—H17	0.9500
C1—H1B	0.9900	C18—C19	1.392 (2)
C2—C3	1.519 (3)	C18—H18	0.9500
C2—H2A	0.9900	C19—H19	0.9500
C2—H2B	0.9900	C20—H20A	0.9800
C3—C4	1.525 (3)	C20—H20B	0.9800
C3—H3A	0.9900	C20—H20C	0.9800
C3—H3B	0.9900	C22—H22	0.9500
C4—C5	1.527 (2)	C24—H24A	0.9800
C4—H4A	0.9900	C24—H24B	0.9800
C4—H4B	0.9900	C24—H24C	0.9800
C5—C6	1.531 (2)	C23—H23A	0.9800
C5—H5A	0.9900	C23—H23B	0.9800
C5—H5B	0.9900	C23—H23C	0.9800
C7—C8	1.479 (2)		
			
C9—S1—C20	102.83 (8)	C8—C9—S1	120.24 (11)
C22—N4—C23	123.6 (2)	C11—C10—C9	121.56 (14)
C22—N4—C24	118.59 (19)	C11—C10—H10	119.2
C23—N4—C24	117.7 (2)	C9—C10—H10	119.2
C13—N1—C6	122.09 (13)	C10—C11—C12	119.13 (14)
C13—N1—H11	117.5 (13)	C10—C11—C14	119.37 (13)
C6—N1—H11	112.7 (13)	C12—C11—C14	121.38 (14)
C7—N2—C6	124.20 (13)	C11—C12—C13	120.40 (14)
C7—N2—H21	114.8 (13)	C11—C12—C21	121.94 (14)
C6—N2—H21	118.3 (13)	C13—C12—C21	117.50 (13)
C2—C1—C6	113.17 (14)	N1—C13—C8	120.12 (13)
C2—C1—H1A	108.9	N1—C13—C12	120.04 (14)
C6—C1—H1A	108.9	C8—C13—C12	119.68 (13)
C2—C1—H1B	108.9	C15—C14—C19	118.98 (15)
C6—C1—H1B	108.9	C15—C14—C11	118.66 (14)
H1A—C1—H1B	107.8	C19—C14—C11	122.36 (14)
C3—C2—C1	111.36 (15)	C16—C15—C14	120.51 (16)
C3—C2—H2A	109.4	C16—C15—H15	119.7
C1—C2—H2A	109.4	C14—C15—H15	119.7
C3—C2—H2B	109.4	C17—C16—C15	120.32 (17)
C1—C2—H2B	109.4	C17—C16—H16	119.8
H2A—C2—H2B	108.0	C15—C16—H16	119.8
C2—C3—C4	111.08 (15)	C16—C17—C18	119.89 (16)
C2—C3—H3A	109.4	C16—C17—H17	120.1
C4—C3—H3A	109.4	C18—C17—H17	120.1
C2—C3—H3B	109.4	C17—C18—C19	120.19 (16)
C4—C3—H3B	109.4	C17—C18—H18	119.9
H3A—C3—H3B	108.0	C19—C18—H18	119.9
C3—C4—C5	110.48 (15)	C18—C19—C14	120.10 (16)
C3—C4—H4A	109.6	C18—C19—H19	120.0
C5—C4—H4A	109.6	C14—C19—H19	120.0
C3—C4—H4B	109.6	S1—C20—H20A	109.5
C5—C4—H4B	109.6	S1—C20—H20B	109.5
H4A—C4—H4B	108.1	H20A—C20—H20B	109.5
C4—C5—C6	111.32 (14)	S1—C20—H20C	109.5
C4—C5—H5A	109.4	H20A—C20—H20C	109.5
C6—C5—H5A	109.4	H20B—C20—H20C	109.5
C4—C5—H5B	109.4	N3—C21—C12	175.83 (16)
C6—C5—H5B	109.4	O2—C22—N4	126.4 (2)
H5A—C5—H5B	108.0	O2—C22—H22	116.8
N1—C6—N2	106.52 (12)	N4—C22—H22	116.8
N1—C6—C5	111.29 (13)	N4—C24—H24A	109.5
N2—C6—C5	110.69 (13)	N4—C24—H24B	109.5
N1—C6—C1	107.40 (13)	H24A—C24—H24B	109.5
N2—C6—C1	110.23 (13)	N4—C24—H24C	109.5
C5—C6—C1	110.60 (13)	H24A—C24—H24C	109.5
O1—C7—N2	121.46 (14)	H24B—C24—H24C	109.5
O1—C7—C8	121.36 (14)	N4—C23—H23A	109.5
N2—C7—C8	116.94 (14)	N4—C23—H23B	109.5
C9—C8—C13	119.62 (13)	H23A—C23—H23B	109.5
C9—C8—C7	122.55 (14)	N4—C23—H23C	109.5
C13—C8—C7	117.44 (13)	H23A—C23—H23C	109.5
C10—C9—C8	119.49 (14)	H23B—C23—H23C	109.5
C10—C9—S1	120.26 (11)		
			
C6—C1—C2—C3	−52.6 (2)	C9—C10—C11—C12	−2.9 (2)
C1—C2—C3—C4	54.9 (2)	C9—C10—C11—C14	173.20 (14)
C2—C3—C4—C5	−57.9 (2)	C10—C11—C12—C13	2.9 (2)
C3—C4—C5—C6	58.0 (2)	C14—C11—C12—C13	−173.08 (14)
C13—N1—C6—N2	37.8 (2)	C10—C11—C12—C21	178.32 (14)
C13—N1—C6—C5	−82.94 (18)	C14—C11—C12—C21	2.3 (2)
C13—N1—C6—C1	155.88 (15)	C6—N1—C13—C8	−18.7 (2)
C7—N2—C6—N1	−39.4 (2)	C6—N1—C13—C12	165.86 (14)
C7—N2—C6—C5	81.73 (18)	C9—C8—C13—N1	−177.79 (14)
C7—N2—C6—C1	−155.59 (15)	C7—C8—C13—N1	−4.8 (2)
C4—C5—C6—N1	−173.96 (13)	C9—C8—C13—C12	−2.4 (2)
C4—C5—C6—N2	67.79 (16)	C7—C8—C13—C12	170.62 (14)
C4—C5—C6—C1	−54.67 (18)	C11—C12—C13—N1	175.10 (15)
C2—C1—C6—N1	173.76 (14)	C21—C12—C13—N1	−0.5 (2)
C2—C1—C6—N2	−70.58 (18)	C11—C12—C13—C8	−0.3 (2)
C2—C1—C6—C5	52.15 (18)	C21—C12—C13—C8	−175.91 (14)
C6—N2—C7—O1	−165.03 (15)	C10—C11—C14—C15	−44.1 (2)
C6—N2—C7—C8	20.5 (2)	C12—C11—C14—C15	131.86 (16)
O1—C7—C8—C9	2.4 (3)	C10—C11—C14—C19	135.62 (16)
N2—C7—C8—C9	176.94 (15)	C12—C11—C14—C19	−48.4 (2)
O1—C7—C8—C13	−170.34 (16)	C19—C14—C15—C16	1.2 (2)
N2—C7—C8—C13	4.2 (2)	C11—C14—C15—C16	−179.02 (15)
C13—C8—C9—C10	2.5 (2)	C14—C15—C16—C17	−0.7 (3)
C7—C8—C9—C10	−170.18 (14)	C15—C16—C17—C18	−0.5 (3)
C13—C8—C9—S1	−178.45 (12)	C16—C17—C18—C19	1.2 (3)
C7—C8—C9—S1	8.9 (2)	C17—C18—C19—C14	−0.6 (3)
C20—S1—C9—C10	−6.48 (15)	C15—C14—C19—C18	−0.6 (2)
C20—S1—C9—C8	174.42 (13)	C11—C14—C19—C18	179.68 (15)
C8—C9—C10—C11	0.2 (2)	C23—N4—C22—O2	178.2 (2)
S1—C9—C10—C11	−178.91 (12)	C24—N4—C22—O2	0.9 (3)

### Hydrogen-bond geometry (Å, °)

**Table:**

*D*—H···*A*	*D*—H	H···*A*	*D*···*A*	*D*—H···*A*
N1—H11···N3^i^	0.838 (10)	2.328 (14)	3.120 (2)	157.96 (3)
N2—H21···O1^ii^	0.886 (10)	2.044 (18)	2.927 (3)	174.94 (3)

Symmetry codes: (i) −*x*+2, −*y*+1, −*z*+1; (ii) −*x*+1, −*y*+1, −*z*.
